# Chinese Herbal Medicines as Natural Alternative Products to Antibiotics in Weaned Piglets through Intestinal Microbiota Regulation

**DOI:** 10.3390/ijms252011034

**Published:** 2024-10-14

**Authors:** Che-Hsuan Wang, Kou-Toung Chung, Li-Yu Su, Wan-Jhen Wu, Pei-Hwa Wang, Ming-Chung Lee, Szu-Chuan Shen, Chung-Hsin Wu

**Affiliations:** 1 School of Life Science, National Taiwan Normal University, Taipei 106, Taiwan; mrgigi1218@gmail.com (C.-H.W.); scs@ntnu.edu.tw (S.-C.S.); 2 Department of Health Nutrition and Chemical Engineering, Army Academy of ROC, Taoyuan City 320, Taiwan; chungkoutoung@gmail.com; 3 Department of Physiology, College of Medicine, National Taiwan University, Taipei 106, Taiwan; julia10025@gmail.com; 4 Graduate Program for Nanotechnology, Department of Materials Science and Engineering, National Yang Ming Chiao Tung University, Hsinchu 300, Taiwan; efgy78@gmail.com; 5 Department of Animal Science and Technology, National Taiwan University, Taipei 106, Taiwan; demonwang@ntu.edu.tw; 6 Brion Research Institute of Taiwan, New Taipei City 231, Taiwan; mileslee@sunten.com.tw

**Keywords:** natural products, Chinese herbal medicine, feed additives, gut microbiota, oxidative stress, inflammation, apoptosis

## Abstract

During the growth process of weaned piglets, digestive problems such as gastrointestinal disorders and diarrhea are common. Farmers usually use antibiotics to help piglets grow smoothly. However, the overuse of antibiotics can lead to antibiotic resistance issues. Therefore, this study chose to use plant extracts as feed additives to explore their potential as alternatives to antibiotics. Additionally, Tilmicosin was used as the antibiotic because it is widely used in treating respiratory infections in piglets. Since traditional Chinese medicine often uses natural products, we selected Guizhi Li-Zhong (GLZ) extract as an alternative to antibiotics. The experiment involved 126 piglets, each 4 weeks old, which were randomly assigned to one of four groups: the sham group (basal diet without supplements, 10.3 ± 0.4 kg, *n* = 31), the low-dose GLZ group (basal diet with 0.05% GLZ, 10.9 ± 0.4 kg, *n* = 32), the regular-dose GLZ group (basal diet with 0.2% GLZ, 10.6 ± 0.4 kg, *n* = 32), and the regular-dose Tilmicosin antibiotic group (basal diet with 0.2% Tilmicosin, 10.2 ± 0.3 kg, *n* = 31). We recorded and compared the survival rate, growth rate, feed conversion ratio, and diarrhea incidence among four groups of weaned piglets from the 4th to the 10th weeks of age. Then, we examined the oxidative stress, inflammation, and apoptosis in small intestine tissue (jejunum and ileum) through immunohistochemistry and Western blot and compared the gut microbiota in large intestine tissue (colon and rectum) through a next-generation sequencing (NGS) analysis. Our results showed that weaned piglets supplemented with 0.05% and 0.2% GLZ had better survival rates, growth rates (*p* < 0.01), and feed conversion ratios (*p* < 0.01) compared to those receiving sham treatment. Even weaned piglets supplemented with 0.2% GLZ performed better than those supplemented with 0.2% Tilmicosin antibiotics (*p* < 0.05). Furthermore, the incidence of diarrhea and small intestine injury (indicated by oxidative stress-, inflammation-, and apoptosis-related proteins) in piglets supplemented with 0.05% and 0.2% GLZ was lower than in piglets receiving sham treatment (*p* < 0.05). Even piglets supplemented with 0.2% GLZ had less injury than those supplemented with 0.2% Tilmicosin antibiotics (*p* < 0.05). The NGS results further showed that GLZ treatment significantly improved beneficial bacteria in weaned piglets (*p* < 0.05), while antibiotic treatment reduced beneficial bacteria (*p* < 0.05). In summary, we recommend adding GLZ to the feed as an alternative to antibiotics. This not only effectively reduces intestinal damage but also improves the gut microbiota, thereby promoting the growth of weaning piglets.

## 1. Introduction

Weaning is a critical stage that affects the growth and development of piglets [1,2]. At weaning, piglets often encounter numerous environmental stressors, such as dietary changes and bacterial and viral infections, leading to dyspepsia, an increased incidence of diarrhea, and slow growth [3,4,5,6]. Consequently, antibiotics are frequently used in feed as growth promoters in weaned piglets and as treatments for gastrointestinal diseases [7,8,9]. While antibiotics as animal feed supplements can effectively inhibit pathogenic bacteria, prevent the growth and reproduction of microorganisms, and improve poor animal growth, their use may lead to antibiotic residues and the risk of bacterial resistance. Moreover, when antibiotics eliminate bacteria, they also kill the original probiotics in the digestive tract [10]. Therefore, it is crucial to develop alternative antibiotic feed additives to alleviate gastrointestinal diseases in piglets. Additionally, weaning can easily cause an imbalance in the intestinal bacteria of piglets, reducing the number of beneficial bacteria and increasing the likelihood of digestive tract diseases [11]. Many studies have found that extracts of traditional Chinese medicine (TCM) have properties against pathogenic microorganisms in the gastrointestinal tract [12,13,14,15] and can reduce gastrointestinal damage by increasing intestinal probiotics [16]. We believe that TCM is very suitable as an alternative to antibiotics for animal feed additives.

In the traditional Chinese Pharmacopoeia, Li-Zhong Tang (LZT) is a renowned TCM formula composed of ginseng, dried ginger, atractylodes, and licorice. LZT has been widely used in the prevention and treatment of gastrointestinal diseases, such as gastric ulcers, duodenal ulcers, and chronic gastritis, due to its anti-inflammatory, antioxidant, immunomodulatory, and gastrointestinal mucosa-repairing effects [17]. Additionally, LZT is often taken with Guizhi. Guizhi decoction, used in China for over 1800 years, is widely employed for respiratory tract infections [18]. GLZ contains ingredients such as ginseng and dried ginger, with the active components of 6-gingerol and 6-shogaol, which have anti-inflammatory properties that help reduce gastrointestinal inflammation and promote better digestion and nutrient absorption. Meanwhile, the licorice, with the active components of liquiritin and glycyrrhizin, has antioxidant effects that help reduce oxidative stress in animals, thereby improving overall health and growth performance. Therefore, GLZ is considered a potential alternative to antibiotics. We combined LZT and Guizhi into Guizhi Li-Zhong Tang (GLZ), which has shown a significant effect in alleviating upper respiratory tract infections, colds, flu, and pneumonia [19]. We observed that weaned piglets suffering from pneumonia had a high expression of angiotensin-converting enzyme 2 (ACE-2) receptors in their upper respiratory tract and alveolar tissue. ACE-2 is a key receptor for the severe acute respiratory syndrome-related coronavirus 2 (SARS-CoV-2) [20]. We further found that GLZ can reduce the expression of ACE-2 receptors in the upper respiratory tract and alveolar tissue of weaned piglets [21]. Therefore, we believe that the effectiveness of GLZ in alleviating respiratory infections and pneumonia in weaned piglets may be partly due to the reduction in ACE-2 receptor expression in the upper respiratory tract and alveolar tissue.

In this study, GLZ was utilized as an animal feed additive, which has significantly enhanced the health and growth rate in weaned piglets. Also, GLZ was used to compare the impacts of antibiotics on the growth performance and intestinal microbiota in weaned piglets. It is possible that GLZ can be considered a viable alternative to antibiotics in animal feed additives.

## 2. Results

### 2.1. Chromatographic Fingerprint Analysis of GLZ

GLZ primarily consists of Guizhi and Lizhong Tang. The main TCM ingredients in GLZ are Guizhi, atractylodes, ginseng, ginger, and licorice. For an HPLC analysis, the standards are typically pure compounds that match the bioactive markers we are looking to identify and quantify. GLZ might be better separated in a liquid phase (HPLC) rather than a gas phase (GC-MS).

**Figure 1:**
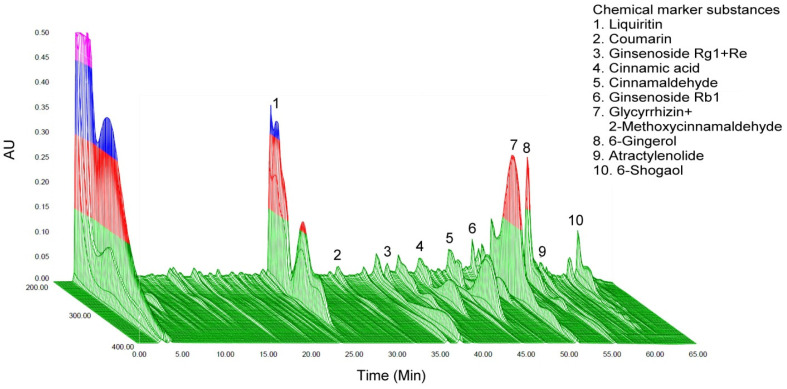
Chromatographic fingerprints of the dietary herbal formula of GLZ. Bioactive marker substances of GLZ are listed on the upper right and were qualitatively determined within 65 min by 3D HPLC. AU, arbitrary perfusion unit.

Figure 1 displays the 3D chromatographic fingerprint of GLZ. The bioactive marker substances in Guizhi include coumarin, cinnamic acid, cinnamic aldehyde, and 2-methoxycinnamaldehyde; Lizhong Tang’s bioactive markers are liquiritin and ginsenoside Rg1+Re, ginsenoside Rb1, glycyrrhizin, 6-gingerol, atractyloid III, and 6-shogaol.

### 2.2. Antioxidant Capacity and Caco-2 Cell Viability of GLZ Treatment

**Figure 2:**
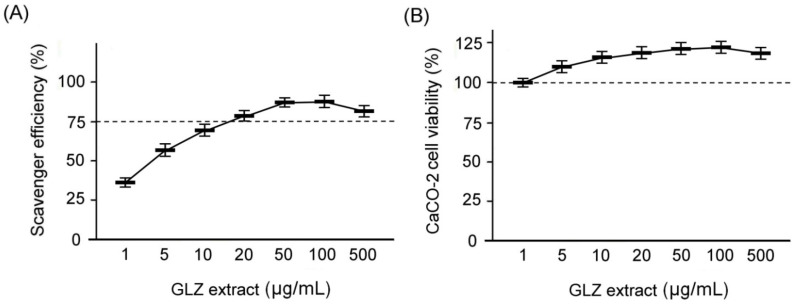
Antioxidant capacity and cytotoxicity of GLZ treatment. (**A**) DPPH free radical method under 1–500 μg/mL GLZ treatments. Dotted line indicates 75% of free radical scavenging activity. (**B**) Quantified Caco-2 cell viability under 1–500 μg/mL GLZ treatments by MTT assay. Dotted line indicates 100% of Caco-2 cell viability. Data are shown as the mean ± SEM, and the sample number must be at least 3 times for each GLZ treatment.

We determined the antioxidant capacity of GLZ using a DPPH assay as in Figure 2A. Our results show that the free radical scavenging activity exceeds 50% when the GLZ treatment concentration ranges between 5 and 100 μg/mL. However, when the GLZ concentration reaches 500 μg/mL, the free radical scavenging activity begins to exhibit a decreasing trend. These results demonstrate that GLZ has a very high antioxidant capacity at appropriate concentrations and can effectively eliminate free radical damage. Next, we assessed the cytotoxicity of GLZ in Caco-2 cells using an MTT assay as in Figure 2B. Our findings reveal that the cell viability of Caco-2 cells treated with GLZ at a concentration of 5–500 μg/mL is higher than that of Caco-2 cells treated with GLZ at 1 μg/mL. The results indicate that GLZ treatment has relatively low cytotoxicity on Caco-2 cells. Based on the results in Figure 1, we posit that GLZ is suitable as an alternative antibiotic animal feed additive for weaned piglets due to its high antioxidant capacity and low cytotoxicity properties.

### 2.3. Survival Rate, Growth Rate, and Feed Conversion Ratio among Four Groups of Weaned Piglets

The survival rate, growth rate, and feed conversion ratio were recorded and compared among the weaned piglets with SH, LD, RD, and RT treatments, as presented in Table 1, Table 2, Table 3 and Table 4. Table 1 illustrates the growth rates among the weaned piglets with SH, LD, RD, and RT treatments from the 4th to the 10th weeks of age. At the 10th week of age, the survival rates of the weaned piglets were 71% for the SH group, 78% for the LD group, 91% for the RD group, and 81% for the RT group. The result shows that the 0.2% GLZ addition treatment exhibits the best survival rate for weaned piglets, surpassing the survival rate of the same concentration of the antibiotic 0.2% Tilmicosin addition. Therefore, we propose that GLZ should be used as an alternative to antibiotics as an animal feed additive.

Table 2 and Table 3 show the average body weight and daily body weight gain among the weaned piglets with SH, LD, RD, and RT treatments from the 4th to the 10th weeks of age. In Table 2, the weaned piglets with SH, LD, RD, and RT treatments at the 4th week of age exhibited no significant weight differences (*p* > 0.05). By the 10th week of age, the average body weight of the weaned piglets was 27.4 ± 1.1 kg for the SH group, 29.6 ± 1.1 kg for the LD group, 32.1 ± 0.9 kg for the RD group, and 30.9 ± 1.0 kg for the RT group. All the weaned piglets at the 10th week of age exhibited a significant weight gain compared to those at the 4th week of age (*p* < 0.01). Additionally, the weaned piglets with LD, RD, RT treatments at the 10th week of age showed a significant weight gain compared to those with SH treatment (*p* < 0.01–0.05).

We further calculated the average daily body weight gain among the weaned piglets with SH, LD, RD, and RT treatments from the 4th to the 10th weeks of age, as presented in Table 3. All the weaned piglets with SH, LD, RD, and RT treatments at the 4th week of age exhibited no differential change in daily body weight gain (*p* > 0.05). At the 6th and 8th weeks of age, the daily body weight gain of the weaned piglets was 0.34 ± 0.07 and 0.37 ± 0.09 kg for the SH group, 0.39 ± 0.09 and 0.41 ± 0.08 kg for the LD group, 0.47± 0.08 and 0.52 ± 0.08 kg for the RD group, and 0.43 ± 0.08 and 0.44 ± 0.07 kg for the RT group. The weaned piglets with LD, RD, and RT treatments at the 6th and 8th weeks of age exhibited a significant daily body weight gain in comparison with those with SH treatment (*p* < 0.01–0.05). At the 10th week of age, the daily body weight gain of the weaned piglets was 0.50 ± 0.09 kg for the SH group, 0.56 ± 0.09 kg for the LD group, 0.57 ± 0.10 kg for the RD group, and 0.51 ± 0.10 kg for the RT group. All the weaned piglets at the 10th week of age exhibited a significant daily body weight gain compared to those at the 4th week of age (*p* < 0.01). Moreover, the weaned piglets with LD, RD, and RT treatments at the 10th week of age exhibited a significant daily body weight gain compared to those with SH treatment (*p* < 0.01–0.05).

We converted the body weight of the weaned piglets with SH, LD, RD, and RT treatments into the feed conversion ratio, which is the amount of food a pig needs to convert food into body weight, as shown in Table 4. All the weaned piglets with SH, LD, RD, and RT treatments at 4 weeks of age exhibited no differential change in the feed conversion ratio (*p* > 0.05). At the 6th, 8th, and 10th weeks of age, the feed conversion ratios for the weaned piglets were 31.3 ± 1.6, 32.7 ± 2.9, and 33.4 ± 3.8% for the SH group; 32.8 ± 1.7, 33.8 ± 2.8, and 34.6 ± 3.3% for the LD group; 36.6 ± 1.6, 37.2 ± 2.8, and 38.1 ± 3.0% for the RD group; and 33.1 ± 1.5, 34.1 ± 2.7, and 35.9 ± 3.2% for the RT group. The weaned piglets with LD, RD, and RT treatments at the 6th, 8th, and 10th weeks of age exhibited a significant feed conversion ratio in comparison with those with SH treatment (*p* < 0.01–0.05).

### 2.4. Blood IgA, IgG, and IgE Levels among Four Groups of Weaned Piglets

We also quantified the IgA, IgG, and IgE levels in the blood of piglets with SH, LD, RD, and RT treatments at 10 weeks of age, as detailed in Table 5. IgA is an antibody that plays a crucial role in the immune function of mucous membranes. IgE can trigger a strong immune response in the body, causing symptoms like redness, swelling, heat, and itching. In contrast, IgG is a protective antibody that can block the IgE reactions, balance the body’s immune system, and prevent allergies. The result shows that those weaned piglets with the LD and RD treatments exhibit significantly increased blood IgA and IgG levels but decreased blood IgE levels than those with SH treatment (*p* < 0.01–0.05).

### 2.5. Antioxidant Stress, Inflammation, and Apoptosis in the Small Intestine Tissue among Four Groups of Weaned Piglets

**Figure 3:**
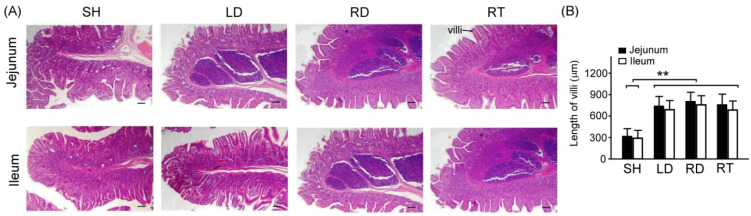
Comparison of tissue morphology of jejunum and ileum among four groups of weaned piglets. (**A**) Representative H&E stains of jejunum and ileum tissue among four groups of weaned piglets. Scale bars = 300 μm. (**B**) Quantified length of the intestinal villi (jejunum and ileum) among four groups of weaned piglets (N = 3 for each group). ** indicates differences *p* < 0.01 between intestinal villi. SH group, sham treatment; LD group, low-dose 0.05% GLZ addition; RD group, regular-dose 0.2% GLZ addition; and RT group, regular-dose antibiotic 0.2% Tilmicosin addition.

We used H&E staining to examine the tissue morphology of the jejunum and ileum among four groups of weaned piglets, as shown in Figure 3A. The results indicate that the quantified length of the intestinal villi in the weaned piglets with SH treatment were significantly shorter and more degenerate than that in the weaned piglets with LD, RD, and RT treatments (Figure 3B, SH vs. LD, RD, and RT, *p* < 0.01). Since intestinal villi are responsible for nutrient absorption, this finding may explain why the weaned piglets with SH treatment exhibited a poorer growth rate and feed conversion ratio than those with LD, RD, and RT treatments.

**Figure 4:**
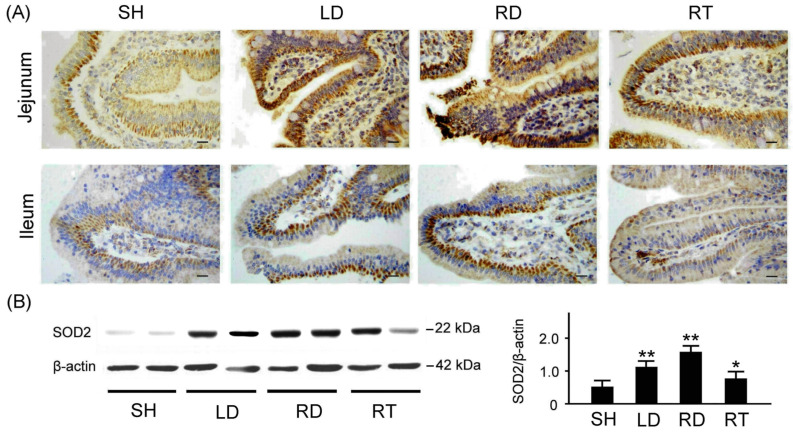
Comparison of antioxidant-related SOD2 expressions in the small intestine tissue among four groups of weaned piglets. (**A**) Representative IHC stains of SOD2 expressions in jejunum and ileum tissue among weaned piglets with SH, LD, RD, and RT treatments. SOD2 expressions are marked with dark brown color. Scale bars = 300 μm. (**B**) Western blotting expressions of SOD2 in the small intestine tissue among weaned piglets with SH, LD, RD, and RT treatments. Right bar chart shows quantified SOD2 expressions relative to β-actin in the small intestine tissue among four groups of weaned piglets (*n* = 3 for each group). SH group, sham treatment; LD group, low-dose 0.05% GLZ addition; RD group, regular-dose 0.2% GLZ addition; and RT group, regular-dose antibiotic 0.2% Tilmicosin addition. Data are shown as the mean ± SEM (** *p* < 0.01 and * *p* < 0.05 compared with the SH group; one-way ANOVA followed by Student–Newman–Keuls multiple comparison posttest).

We utilized immunohistochemistry (IHC) and Western blot techniques to compare the oxidative stress, inflammation, and apoptosis in the small intestine tissue among piglets at 10 weeks of age with SH, LD, RD, and RT treatments. To assess antioxidant capacity, we examined antioxidant-related SOD2 expressions in the small intestine tissue among piglets with SH, LD, RD, and RT treatments, as depicted in Figure 4. As shown in Figure 4A, piglets with SH treatment exhibited obviously reduced SOD2 expressions in the jejunum and ileum tissue than those with LD, RD, and RT treatments. Figure 4B shows the quantified SOD2 expressions relative to β-actin in the small intestine tissue that were 0.52 ± 0.19 for the SH group, 1.10 ± 0.18 for the LD group, 1.55 ± 0.17 for the RD group, and 0.76 ± 0.21 for the RT group. The result shows that the weaned piglets with SH treatment exhibit significantly lower SOD2 expressions in the small intestine tissue compared to those with LD, RD, and RT treatments (*p* < 0.01–0.05).

**Figure 5:**
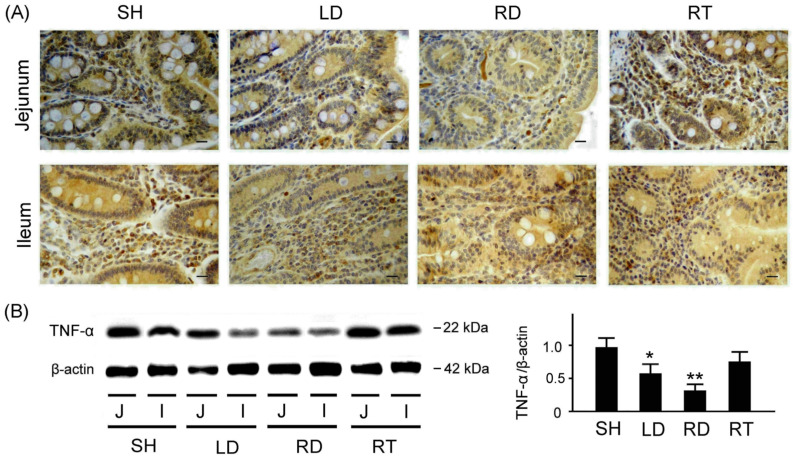
Comparison of inflammation-related TNF-α expressions in the small intestine tissue among four groups of weaned piglets. (**A**) Representative IHC stains of TNF-α expressions in jejunum and ileum tissue among weaned piglets with SH, LD, RD, and RT treatments. TNF-α expressions are marked with dark brown color. Scale bars = 300 μm. (**B**) Western blotting expressions of TNF-α in the small intestine tissue among weaned piglets with SH, LD, RD, and RT treatments. Right bar chart shows quantified TNF-α expressions relative to β-actin in the small intestine tissue among four groups of weaned piglets (*n* = 3 for each group). SH group, sham treatment; LD group, low-dose 0.05% GLZ addition; RD group, regular-dose 0.2% GLZ addition; and RT group, regular-dose antibiotic 0.2% Tilmicosin addition. (** *p* < 0.01 and * *p* < 0.05 compared with the SH group; one-way ANOVA followed by Student–Newman–Keuls multiple comparison posttest).

To assess inflammation, we evaluated the inflammation-related TNF-α expressions in the small intestine tissue among piglets with SH, LD, RD, and RT treatments (Figure 5). According to Figure 5A, the piglets with SH and RT treatments exhibited significantly increased TNF-α expressions in the jejunum and ileum tissue compared to those with LD and RD treatments. Figure 5B presents the quantified TNF-α expressions relative to the β-actin in the small intestine tissue, with values of 0.96 ± 0.14 for the SH group, 0.59 ± 0.13 for the LD group, 0.32 ± 0.08 for the RD group, and 0.91 ± 0.14 for the RT group. These results show that the weaned piglets with SH and RT treatments exhibit significantly higher TNF-α expressions in the small intestine tissue compared to those with LD and RD treatments (*p* < 0.01–0.05).

**Figure 6:**
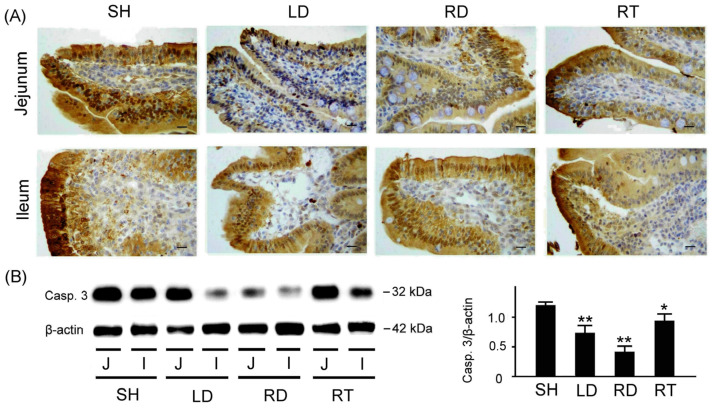
Comparison of apoptosis-related caspase 3 expressions in the small intestine tissue among four groups of weaned piglets. (**A**) Representative IHC stains of caspase 3 expressions in jejunum and ileum tissue among weaned piglets with SH, LD, RD, and RT treatments. Caspase 3 expressions are marked with dark brown color. Scale bars = 300 μm. (**B**) Western blotting expressions of caspase 3 in the small intestine tissue among weaned piglets with SH, LD, RD, and RT treatments. Right bar chart shows quantified caspase 3 expressions relative to β-actin in the small intestine tissue among four groups of weaned piglets (*n* = 3 for each group). SH group, sham treatment; LD group, low-dose 0.05% GLZ addition; RD group, regular-dose 0.2% GLZ addition; and RT group, regular-dose antibiotic 0.2% Tilmicosin addition. (** *p* < 0.01 and * *p* < 0.05 compared with the SH group; one-way ANOVA followed by Student–Newman–Keuls multiple comparison posttest).

To compare apoptosis, we examined apoptosis-related caspase 3 expressions in the small intestine tissue among piglets with SH, LD, RD, and RT treatments, as shown in Figure 6. As indicated in Figure 6A, piglets with SH treatment displayed markedly increased caspase 3 expressions in the jejunum and ileum tissue compared to those with LD, RD, and RT treatments. Figure 6B shows the quantified caspase 3 expressions relative to β-actin in the small intestine tissue: 19 ± 0.06 for the SH group, 0.72 ± 0.12 for the LD group, 0.41 ± 0.09 for the RD group, and 0.89 ± 0.12 for the RT group. The result shows that weaned piglets with SH treatments exhibit significantly higher caspase 3 in the small intestine tissue than those with LD, RD, and RT treatments (*p* < 0.01–0.05).

### 2.6. Gut Microbiota Diversity in the Large Intestine Tissue among Four Groups of Weaned Piglets

**Figure 7:**
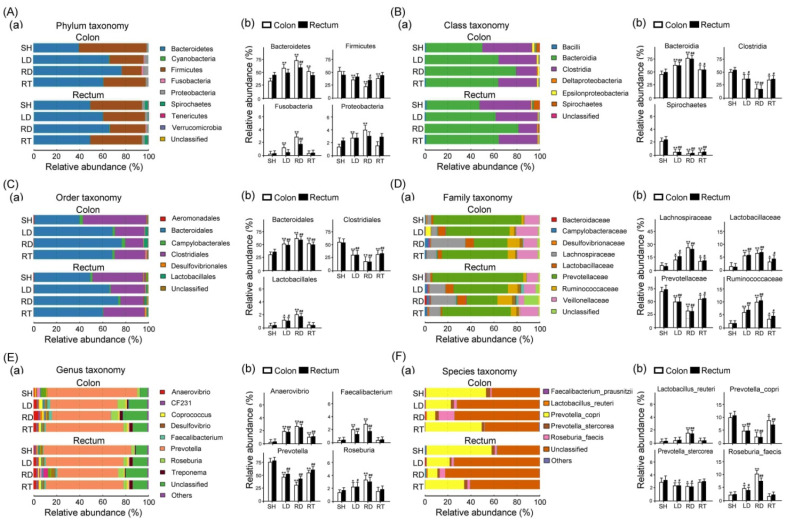
Gut microbial community structure in the colon and rectum of the large intestine tissue among four groups of weaned piglets. The microbial community bar plot in the colon and rectum tissue of weaned piglets in (**A**) phylum level (a) relative abundance of most abundant OTUs in phyla level of each sample, and (b) quantified relative abundance of Bacteroidetes, Firmicutes, Fusobacteria, and Proteobacteria in phyla level of each sample; (**B**) Class level (a) relative abundance of most abundant OTUs in class level of each sample, and (b) quantified relative abundance of Bacteroidia, Clostridia, and Spirochaetes in class level of each sample; (**C**) Order level (a) relative abundance of most abundant OTUs in order level of each sample, and (b) quantified relative abundance of Bacteroidales, Clostridiales, and Lactobacillales in order level of each sample; (**D**) Family level (a) relative abundance of most abundant OTUs in family level of each sample, and (b) quantified relative abundance of Lachnospiraceae, Lactobacillaceae, Prevotellaceae, and Ruminococcaceae, in family level of each sample; (**E**) Genus level (a) relative abundance of most abundant OTUs in genus level of each sample, and (b) quantified relative abundance of Anaerovibrio, Faecalibacterium, Prevotella, and Roseburia in genus level of each sample; (**F**) Species level (a) relative abundance of most abundant OTUs in species level of each sample, and (b) quantified relative abundance of *Lactobacillus_reuteri*, *Prevotella_copri*, *Prevotella_stercorea,* and *Roseburia_faecis*. SH group, sham treatment; LD group, low-dose 0.05% GLZ addition; RD group, regular-dose 0.2% GLZ addition; and RT group, regular-dose antibiotic 0.2% Tilmicosin addition. (*n* = 3 for each group, ** *p* < 0.01 and * *p* < 0.05 compared with the SH group; ^##^ *p* < 0.01 and ^#^ *p* < 0.05 compared with the colon group, two-way ANOVA followed by Student–Newman–Keuls multiple comparison posttest).

To compare the gut microbiome in the colon and rectum of large intestine tissue among piglets at the 10th week of age with SH, LD, RD, and RT treatments, we conducted a next-generation sequencing (NGS) analysis. Figure 7 illustrates histograms depicting the community structure of the gut microbiota, revealing the microbial species and their relative abundance. In Figure 7, the gut microbiome by phylum in the colon and rectum of large intestine tissue of the weaned piglets contained Actinobacteria, Bacteroidetes, Firmicutes, Fusobacteria, and Proteobacteria. 

The most abundant phyla were Bacteroidetes, Firmicutes, and Proteobacteria (Figure 7A(a)). Our data show that the gut microbiome in the large intestine tissue of the weaned piglets with LD and RD treatments exhibited a significantly increased relative abundance of Bacteroidetes, Fusobacteria, and Proteobacteria but a decreased relative abundance of Firmicutes compared to those with SH treatment (Figure 7A(b)). In Figure 7B, the gut microbiome by Class in the colon and rectum of the large intestine tissue of the weaned piglets contained Bacilli, Bacteroidia, Clostridia, Deltaproteobacteria, Epsilonproteobacteria, and Spirochaetes. The most abundant Classes were Bacteroidia and Clostridia (Figure 7B(a)). Those gut microbiomes in the large intestine tissue of the weaned piglets with LD, RD, and RT treatments exhibited a significantly increased relative abundance of Bacteroidia but a decreased relative abundance of Clostridia and Spirochaetes than those of the weaned piglets with SH treatment (Figure 7B(b)). In Figure 7C, the gut microbiome by Order in the colon and rectum of large intestine tissue of the weaned piglets contained Aeromonadales, Bacteroidales, Campylobacterales, Clostridiales, Desulfovibrionales, and Lactobacillales. The most abundant Orders were Bacteroidales and Clostridiales (Figure 7C(a)). Those gut microbiomes in the large intestine tissue of the weaned piglets with LD, RD, and RT treatments exhibited a significantly increased relative abundance of Bacteroidales and Lactobacillales but a decreased relative abundance of Clostridiales than those with SH treatment (Figure 7C(b)). In Figure 7D, the gut microbiome by Family in the colon and rectum of the large intestine tissue of the weaned piglets contained Bacteroidaceae, Campylobacteraceae, Desulfovibrionaceae, Lachnospiraceae, Lactobacillaceae, Prevotellaceae, Ruminococcaceae, and Veillonellaceae. The most abundant Families were Lachnospiraceae, Prevotellaceae, Ruminococcaceae, and Veillonellaceae (Figure 7D(a)). Those gut microbiomes in the large intestine tissue of the weaned piglets with LD, RD, and RT treatments exhibited a significantly increased relative abundance of Lachnospiraceae, Lactobacillaceae, and Ruminococcaceae but a decreased relative abundance of Prevotellaceae than those with SH treatment (Figure 7D(b)). In Figure 7E, the gut microbiome by Genus in the colon and rectum of the large intestine tissue of the weaned piglets contained Anaerovibrio, CF231, Coprococcus, Desulfovibrio, Faecalibacterium, Prevotella, Roseburia, and Treponema. The most abundant Genera were Prevotella and Roseburia (Figure 7E(a)). Those gut microbiomes in the large intestine tissue of the weaned piglets with LD, RD, and RT treatments exhibited a remarkably increased relative abundance of Anaerovibrio, Faecalibacterium, and Roseburia but a decreased relative abundance of Prevotella than those with SH treatment (Figure 7E(b)). Finally, in Figure 7F, the gut microbiome by Species in the colon and rectum of the large intestine tissue of the weaned piglets contained *Faecalibacterium_ prausnitzii*, *Lactobacillus_reuteri*, *Prevotella_copri*, *Prevotella_stercorea,* and *Roseburia_faecis*. The most abundant Species were *Prevotella_copri* and *Roseburia_faecis* (Figure 7F(a)). Those gut microbiomes in the large intestine tissue of the weaned piglets with LD, RD, and RT treatments exhibited a significantly increased relative abundance of *Lactobacillus_reuteri* and *Roseburia_faecis* but a decreased relative abundance of *Prevotella_copri* and *Prevotella_stercorea* compared to those with SH treatment (Figure 7F(b)).

## 3. Discussion

In this study, we selected an herbal formula, GLZ, as a feed additive for weaned piglets to potentially replace antibiotics. Weaned piglets often suffer from poor digestive adaptation during growth. Typically, owners use antibiotics to ensure smooth growth. However, improper or excessive use of antibiotics may lead to adverse reactions or side effects in pigs, such as antibiotic residues and resistance, which can further affect human and animal health. As suggested from our results, GLZ can promote the number of probiotic bacteria in the intestines of weaned piglets, thereby enhancing immunity and inhibiting the growth of pathogenic bacteria. This result is different from the use of antibiotics as feed additives, which not only inhibit the growth of pathogenic bacteria but also inhibit the growth of probiotic bacteria in the gastrointestinal tract.

Weaned piglets growing in the winter are often susceptible to colds due to low temperatures, which can even lead to pneumonia and, in severe cases, death. Therefore, we included TCMs of Guizhi and Li-Zhong Tang in a specific ratio in GLZ as an animal feed additive. Cinnamaldehyde, the main component of Guizhi, has antioxidant, anti-inflammatory, and antibacterial properties [22,23] and is also an NF-κB inhibitor [24]. NF-κB regulates the expression of cytokines and chemokines (such as TNF-α), as well as pro-apoptotic and apoptotic proteins [25,26]. In addition, components of Guizhi, such as coumarin and cinnamic acid, have strong antioxidant, anti-inflammatory [27], and neuroprotective capabilities [28]. Li-Zhong Tang comprises four herbal medicines: ginseng, dried ginger, atractylodes, and licorice. Ginseng contains ginsenoside Rg1+Re, which reduces oxidative stress and alleviates cardiomyocyte apoptosis [29]. Ginsenoside Rb1 has antioxidant and anti-inflammatory pharmacological functions and achieves antioxidant and anti-inflammatory effects by weakening NF-κB and MAPK activation [30]. Dried ginger contains 6-gingerol and 6-shogaol, both of which have antioxidant and anti-inflammatory activities [31]. Atractylodes consists of atractyloid III, which treats inflammation-related neurodegenerative diseases by weakening the activity of NF-κB [32]. Licorice contains liquiritin and exhibits antioxidant, anti-inflammatory, and anti-apoptotic protective effects by inhibiting NF-κB and MAPK signaling pathways [33]. Glycyrrhizin, another component of licorice, has antioxidant stress and anti-inflammatory effects and can be used to treat liver diseases [34]. Our previous experiments have shown that GLZ can reduce oxidative stress, inflammation, and apoptosis and inhibit the expression of ACE-2 in the respiratory tract and lung tissue of weaned piglets [19,21], thereby alleviating pneumonia.

Since the small intestine is the primary organ for the digestion and absorption of nutrients, its intact mucosal structure is critical for nutrient absorption and optimal growth [35]. Weaning stress can lead to structural and functional changes in the intestinal mucosa of weaned piglets [36]. Our results indicate that dietary GLZ supplementation can increase the villus height in the small intestine to a level similar to that achieved with antibiotic treatment, indicating an increase in the absorption area of the intestinal mucosa (Figure 3). Furthermore, we observed that the GLZ supplement protects against oxidative stress, inflammation, and apoptosis in the small intestine tissue of weaned piglets (Figure 4, Figure 5 and Figure 6). These results align with the observed improvements in growth performance and diarrhea rate, suggesting that the benefits of GLS may be related to improvements in the structure and function of the small intestine.

The health status and growth rate of weaned piglets are closely related to the gut microbiota [37]. The development of many piglet diseases is accompanied by imbalances in the gut microbiota, including changes in the number and types of bacterial flora. Our study compares the effects of GLZ and antibiotics on the gut microbiota of weaned piglets using NGS. We found that the dietary GLZ supplement modulates gut microbiota. Compared with the sham and antibiotic group, the gut microbiota of weaned piglets with dietary GLZ supplementation shows a significant increase in the relative abundance of Bacteroidetes, Fusobacteria, and Proteobacteria but a decrease in the relative abundance of Firmicutes at the phylum level (Figure 7A). At the Class level, GLZ significantly increases the relative abundance of Bacteroidia but decreases the relative abundance of Clostridia and Spirochaetes (Figure 7B). And the GLZ supplement remarkably increases the relative abundance of Bacteroidales and Lactobacillales but decreases the relative abundance of Clostridiales at the Order level (Figure 7C). In terms of the Family level, the GL significantly increases the relative abundance of Lachnospiraceae, Lactobacillaceae, and Ruminococcaceae but decreases the relative abundance of Prevotellaceae (Figure 7D). Regarding the Genus level, the GLZ increases the relative abundance of Anaerovibrio, Faecalibacterium, and Roseburia but reduces the relative abundance of Prevotella (Figure 7E). For the Species level, the GLZ significantly increases the relative abundance of *Lactobacillus_reuteri* and *Roseburia_faecis* but decreases the relative abundance of *Prevotella_copri* and *Prevotella_stercorea* (Figure 7F). In general, the normal gut microbiota comprises two major phyla, namely, Bacteroidetes and Firmicutes, that represent more than 90% of the total gut microbiota [38]. Most dietary polysaccharides are not digested by the gastrointestinal tract but can be broken down into absorbable metabolites, such as short-chain fatty acids, especially by the phylum Bacteroidetes (Bacteroidia and Bacteroidales) [39]. Because the Firmicutes/Bacteroidetes ratio is frequently cited as a hallmark of metabolic disorders [40], we found that the Firmicutes/Bacteroidetes ratio of weaned piglets with low- and regular-dose GLZ addition is obviously lower than those with sham and regular-dose antibiotic addition. Many studies suggested that the phylum Lactobacillales (Lactobacillaceae and *Lactobacillus_reuteri*) may be helpful in modulating gut microbiota, eliminating infections, and attenuating the gastrointestinal symptoms of enteric colitis, antibiotic-associated diarrhea, irritable bowel syndrome, inflammatory bowel disease, and chronic constipation [41]. Ruminococcaceae can be better adapted to the gut with inflammatory bowel disease through mechanisms of oxidative stress responses and mucus utilization [42]. Roseburia (*Roseburia_faecis*) can produce butyrate in the colon that has been shown to prevent intestinal inflammation and maintain energy homeostasis by producing metabolites [43]. Bacteroidaceae, Faecalibacterium, and Lachnospiraceae have been shown to enhance short-chain fatty acid production and increase butyrate derivatives [44]. Pevotellaceae (Prevotella, *Prevotella_copri*, and *Prevotella_stercorea*) is an abundant member of the human gastrointestinal microbiome, whose relative abundance has curiously been associated with positive and negative impacts on diseases, such as Parkinson’s disease and rheumatoid arthritis [45]. Clostridia (Clostridiales) is considered the most relevant enteropathogen of neonatal piglets [46]. Using HPLC analysis, the main active compounds in GLZ, such as cinnamaldehyde, ginsenosides, 6-gingerol, and glycyrrhizin, were identified. Studies on microbial community correlations found that the presence and concentration of specific compounds are associated with changes in microbial populations. For example, cinnamaldehyde is known for its antibacterial properties and may be linked to an increase in beneficial bacteria like lactobacilli and a decrease in pathogenic bacteria, like clostridia [47]. Ginsenosides, with their antioxidant and anti-inflammatory properties, may support the growth of beneficial bacteria, such as *Faecalibacterium* and *Ruminococcus* [48]. 6-Gingerol and 6-shogaol may be associated with an increase in *Bacteroidetes* and a decrease in *Firmicutes*, improving gut health and reducing inflammation [49]. Glycyrrhizin’s anti-inflammatory effects may be related to an increase in beneficial bacteria like *Lactobacillus reuteri* and a decrease in pathogenic bacteria, like *Prevotella* [50]. Cinnamaldehyde, as an NF-κB inhibitor, may reduce inflammation, creating a more favorable environment for probiotics [51]. Integrating the study results, we find that the presence of specific compounds in GLZ is associated with changes in the gut microbiota and improved health outcomes. For instance, an increase in *Bacteroidetes* and *lactobacilli* is linked to better digestion and reduced inflammation. A decrease in *Firmicutes* and *Clostridium* is associated with reduced metabolic disorders and lower rates of gut infections. To sum up, dietary GLZ supplementation can balance the number of probiotics and pathogenic bacteria, thereby maintaining a healthy intestinal environment.

## 4. Materials and Methods

### 4.1. GLZ Preparation and High-Performance Liquid Chromatography (HPLC) Analysis

In this study, we combined TCMs of Guizhi and Li-Zhong Tang in a specific ratio to create Guizhi Li-Zhong Tang (GLZ), which was used as an animal feed additive. This preparation was obtained from Sun-Ten Pharmaceutical Company (New Taipei City, Taiwan). High-performance liquid chromatography (HPLC) was then employed to analyze the bioactive marker substances in GLZ. The analysis method for GLZ proceeded as follows: GLZ was first dissolved in purified water provided by the Milli-Q Water Purification System (Millipore, MA, USA) with an analytical concentration of 0.4 mg/mL. The solution was then fractionated using acetonitrile and methanol (Burdick & Jackson Korea, Seoul, Republic of Korea). Qualitative determination was performed using HPLC (Thermo Fisher Scientific Inc., Waltham, MA, USA) under selected conditions within a 70 min timeframe. The HPLC analysis conditions were set as follows: the column (Waters/Sunfire RP18, Milford, MA, USA) measured 5 µm and 150 mm × 4.6 mm ID; the mobile phase consisted of 0.05% aqueous trifluoroacetic acid (solution A) and acetonitrile (solution B). Detection was conducted using a photodiode array (λ = 220 nm), and the injection volume was 10 µL. The oven temperature was maintained at 30 °C, and the run time was set to 20 min. To analyze the GLZ by HPLC, the standards for coumarin, cinnamic acid, cinnamic aldehyde, 2-methoxycinnamaldehyde, liquiritin, ginsenoside Rg1+Re, ginsenoside Rb1, glycyrrhizin, 6-gingerol, atractyloid III, and 6-shogaol were used.

### 4.2. Determination of Antioxidant Capacity of GLZ

The antioxidant activity of GLZ was evaluated using the 1,1-diphenyl-2-picryl- hydrazyl (DPPH, D9132, Sigma-Aldrich Co., St. Louis, MO, USA). This assay was performed as follows: GLZ was first diluted with distilled water to prepare solutions at different concentrations (1, 5, 10, 20, 50, 100, and 500 µg/mL) and then we added 100 μL of 1.5 mM/mL DPPH (Sigma-Aldrich Co.) into each well of a 96-well plate, followed by the addition of different concentrations of GLZ to the wells containing DPPH. The mixture was incubated at room temperature for 30 min. Subsequently, the absorbance at 517 nm was measured using a microplate spectrophotometer (μQuant, Biotek Intruments, Inc., Charlotte, VT, USA). For each concentration of GLZ, three separate DPPH determinations were performed. To benchmark the antioxidant function of the GLZ, we also measured the absorbance of blank methanol and L-ascorbic acid (A5960, Sigma-Aldrich) as the controls. The DPPH scavenging activity was calculated using the formula of GLZ (%) = 100 × [(absorbance of GLZ + DPPH) − (absorbance of GLZ blank)]/[(absorbance of DPPH) − (absorbance of methanol)].

### 4.3. Cell Viability Assay of GLZ

We used 3-(4,5-dimethylthiazol-2-yl)-2,5-diphenyltetrazolium bromide (MTT, M5655, Sigma-Aldrich Co., St. Louis, MO, USA) to evaluate the effect of GLZ in the cell viability of Caco-2 cells. The Caco-2 cells (BCRC Number: 60182), which are human colon adenocarcinoma cells, were purchased from the Bioresource Collection and Research Center (BCRC, Hsinchu, Taiwan). The MTT assay was performed as follows: We seeded 1 × 10^5^ cells/mL of Caco-2 cells into a 24-well plate and treated them with different concentrations of GLZ solution (1, 5, 10, 20, 50, 100, and 500 µg/mL). After 24 h of culture, we added 0.5 mg/mL of MTT solution (M5655, Sigma-Aldrich Co., St. Louis, MO, USA) and incubated the cells for an additional 2 h. The supernatant was then removed, and 100 μL/well of DMSO was added. The plates were shaken for 5 min, and the absorbance was measured at 570 nm.

### 4.4. Animal Preparation and Grouping

The experiment involved 126 crossbreed LYD piglets, each 4 weeks old, which were randomly assigned to one of four groups: the sham group (SH, basal diet without supplements, 10.3 ± 0.4 kg, *n* = 31), the low-dose GLZ group (LD, basal diet with 0.05% GLZ, 10.9 ± 0.4 kg, *n* = 32), the regular-dose GLZ group (RD, basal diet with 0.2% GLZ, 10.6 ± 0.4 kg, *n* = 32), and the regular-dose Tilmicosin antibiotic group (RT, basal diet with 0.2% Tilmicosin, 10.2 ± 0.3 kg, *n* = 31). The regular level we used for comparison with the antibiotic Tilmicosin was 0.2%, and the regular level used to define GLZ was also 0.2%. The piglets were reared at Dafeng Ranch (Yunlin County, Taiwan) from the 4th to the 10th week of age under specific pathogen-free conditions, at a constant temperature of 25 °C and with 12 h light/dark cycles. At the age of 10 weeks, three weaned piglets from each group were randomly selected for sacrifice to collect small intestine and large intestine tissue. Our piglet experiments adhered to the 3R principles and the international guidelines for the Care and Use of Laboratory Animals (Permit number: NTNU/Animal Use/No. 109007).

### 4.5. Survival Rate, Growth Rate, and Feed Conversion Ratio Survey

The survival rate, growth rate, and feed conversion ratio among the weaned piglets subjected to SH, LD, RD, and RT treatments were recorded once every two days from the 4th to the 10th week of age. The survival rate was calculated as the percentage of eliminated individuals from the total number of surviving individuals. The growth rate was determined by measuring the daily weight gain of the SH, LD, RD, and RT piglets. The feed conversion ratio, an indicator of how efficiently pigs convert food into body weight, was also measured. A lower feed conversion ratio indicates a more efficient conversion of feed to body weight.

### 4.6. Blood Immunoglobulin Test

At the age of 10 weeks, blood samples were collected from the SH, LD, RD, and RT piglets. The levels of immunoglobulin A (IgA), E (IgE), and G (IgG) in the blood were analyzed using a sandwich ELISA kit (E4472-100 & E4476-100; BioVision, Milpitas, CA, USA). The ELISA tests were performed according to the manufacturer’s instructions using a Multiskan™ GO Microplate Spectrophotometer reader (Thermo Scientific™, Waltham, MA, USA).

### 4.7. Diarrhea Incidence Analysis

The incidence of diarrhea among the SH, LD, RD, and RT piglets was recorded daily from the 4th to the 10th week of age using continuous video image analysis. Diarrhea was defined according to the World Health Organization (WHO) as an increase in the water content of the stool and an increase in the frequency of stools to three times or more times per day. Continuous image analysis can be a valuable tool for monitoring the health of piglets, including detecting signs of diarrhea. Here are some steps and considerations for implementing such a system: Camera Setup: Install high-resolution cameras (Canon VIXIA HF G70 camera, Tokyo, Japan) in the piglet pens to capture continuous video footage. The video capture resolution is 4 K and ensure the cameras cover all areas where the piglets spend time. Image Processing Software: Use Sigmascan pro 5.0 software (SPSS Inc., San Jose, CA, USA) that can analyze video footage in real time. This software should be capable of detecting changes in piglet behavior and physical appearance, such as increased lying down, changes in posture, or visible signs of diarrhea. Behavioral Analysis: Monitor for specific behaviors that may indicate diarrhea, such as increased frequency of lying down, reluctance to move, or changes in feeding and drinking patterns. Physical Signs: Detect physical signs of diarrhea, such as wetness around the tail area or changes in the consistency and color of feces. Image analysis algorithms can be trained to recognize these signs. Data Integration: Combine image analysis data with other health monitoring data, such as feed and water intake, weight changes, and temperature readings. This holistic approach can improve the accuracy of early detection. Machine Learning Models: Develop and train machine learning models to identify patterns associated with diarrhea. These models can learn from historical data to improve their predictive accuracy over time. Alerts and Notifications: Set up the system to send alerts to farm staff when potential signs of diarrhea are detected. This allows for timely intervention and treatment. Regular Calibration: Regularly calibrate and update the image analysis system to ensure its accuracy and reliability. This includes updating the software and retraining the machine learning models as needed.

### 4.8. Histochemistry and Immunohistochemistry Staining

At the 10th week of age, the jejunum and ileum tissues of the small intestine were collected from the four groups of weaned piglets. Morphologically, the jejunum is characterized by a larger diameter, thicker walls, more blood vessels, and a redder color, while the ileum has a thinner diameter, thinner walls, fewer blood vessels, and a lighter color. The small intestine was fixed in 4% formaldehyde and embedded in paraffin. Sections of 5 μm thickness were cut from the jejunum and ileum samples using a microtome and mounted on glass slides for staining. Hematoxylin and eosin (H&E) staining was performed using an H&E kit (Sigma-Aldrich Co., St. Louis, MO, USA) to assess the tissue morphology. In the H&E kit staining, hematoxylin stains cell nuclei purple-blue, eosin stains the extracellular matrix and cytoplasm pink, and other structures appear in different combinations of dark and light tones. Therefore, H&E staining facilitates the easy distinction of nuclear and cytoplasmic parts of cells, providing an overview of tissue cells.

In addition, we employed immunohistochemistry (IHC) staining to examine the protein expression related to antioxidant stress, inflammation, and apoptosis in the jejunum and ileum tissues of the weaned piglets. The IHC staining utilized a heat-induced epitope retrieval method. Tissues sections were stained with antibodies against superoxide dismutase 2 (SOD2; Cell Signaling Technology Inc., Danvers, MA, USA), tumor necrosis factor-α (TNF-α; Cell Signaling Technology Inc.), and caspase 3 (Cell Signaling Technology Inc.) for 1 h at room temperature. IHC detection involved incubating tissue sections with a biotinylated secondary antibody (Novolink polymer detection system 1) at room temperature for 30 min, followed by incubation with avidin-biotin-HRP complex (Novolink polymer detection system 1) for another 30 min. IHC imaging was performed with DAB chromogen (Novolink polymer detection system 1) and counterstaining was carried out with hematoxylin (Novolink polymer detection system 1) following the supplier’s protocol.

### 4.9. Western Blotting

The jejunal and ileal tissue removed from the small intestine were homogenized in a buffer solution. Then, the tissue proteins present in the isolated solution were quantified using a bicinchoninic protein assay kit (Thermo Fisher Scientific Inc.) and separated through polyacrylamide gel electrophoresis (Bionovas Pharmaceuticals Inc., Washington DC, USA). The separated proteins were transferred onto polyvinylidene fluoride membranes (GE Healthcare Life Sciences, Barrington, IL, USA). Antibodies against β-actin (Thermo Fisher Scientific Inc.), SOD2, TNF-α, and caspase 3 (Cell Signaling Technology Inc.) were used. The primary antibodies were detected using appropriate horseradish peroxidase–conjugated secondary antibodies (Santa Cruz Biotechnology Inc. Dallas, Texas, USA). The immunostained samples were visualized using the enhanced chemiluminescence substrate (Millipore, Burlington, MA, USA) and quantified using ImageJ (version 1.48t; National Institutes of Health, Washington, DC, USA).

### 4.10. DNA Extraction of Large Intestine Tissue and Next-Generation Sequencing (NGS)

At 10 weeks of age, colon and rectum tissues from the large intestine were collected from the four groups of weaned piglets. The colon and rectum, belonging to the lower digestive system, have distinct functions: the colon absorbs residual water and electrolytes in the adnominal cavity, while the rectum, connected to the anal sphincter, primarily functions to store feces. After receiving the colon and rectal tissue DNA samples, we checked and constructed a database for qualified samples; recovered the target amplicon fragments; and used T4 DNA polymerase, Klenow DNA polymerase, and T4 PNK to repair the sticky ends formed by the interruption into blunt ends. Thereafter, an alkyl group “A” was added to the 3′ end, enabling the DNA fragment to connect to a special adapter with a “T” alkyl group at the 3′ end. We designed and synthesized a double-index fusion primer containing a sequencing adapter, used genomic DNA as a template, performed fusion primer PCR, and used magnetic beads to screen the target amplicon fragment. Finally, we used qualified databases for cluster preparation and sequencing, utilizing offline data for the corresponding biological information analysis.

After offline data filtering, the low-quality data were removed, leaving high-quality clean data for the subsequent analysis. We spliced the reads into Tags through the overlap relationship between the reads, clustered the Tags into Operational Units (OTUs) under a given similarity, and then compared the OTUs with the database to annotate the species on the OTUs. Based on the OTU and species annotation results, we performed a sample species complexity analysis and inter-group species difference analysis. Then, we performed OTU clustering on the processed Clean Tags and completed the species classification of OTUs by annotating the OTUs. Finally, we used the software USEARCH (v7.0.1090) [52] (Edgar, 2013) to cluster the spliced Tags into OTUs.

### 4.11. Statistical Analysis

The quantified data were expressed as the mean ± standard error. A statistical analysis was conducted using one-way or two-way ANOVA followed by the Student–Newman–Keuls multiple comparison posttest. A *p*-value of < 0.05 was considered significant.

## 5. Conclusions

In this study, we found that the Chinese herbal medicine GLZ can alleviate gastrointestinal disorders and promote better growth in weaned piglets, making it a viable natural alternative to antibiotics. Our results demonstrate that GLZ can effectively alleviate oxidative stress, inflammation, and apoptosis in the small intestinal tissues and obviously improve the intestinal flora in the large intestinal tissues of weaned piglets. Compared to weaned piglets using antibiotics as feed additives, those using GLZ as a feed additive exhibited a higher survival rate, a better growth rate, an improved feed conversion ratio, and enhanced immunity while experiencing lower diarrhea rates. Therefore, we believe that GLZ can indeed serve as a natural alternative product to antibiotics.

## Figures and Tables

**Table 1:** Survival rate (%) among four groups of weaned piglets.

Group/Age	4 Weeks	6 Weeks	8 Weeks	10 Weeks
SH	100% (n = 31)	94% (n = 29)	81% (n = 25)	71% (n = 22)
LD	100% (n = 32)	91% (n = 29)	78% (n = 25)	78% (n = 25)
RD	100% (n = 32)	97% (n = 31)	91% (n = 29)	91% (n = 29)
RT	100% (n = 31)	96% (n = 30)	84% (n = 26)	81% (n = 25)

SH group, sham treatment; LD group, low-dose 0.05% GLZ addition; RD group, regular-dose 0.2% GLZ addition; and RT group, regular-dose antibiotic 0.2% Tilmicosin addition.

**Table 2:** Average body weight (kg) among four groups of weaned piglets.

Group/Age	4 Weeks	6 Weeks	8 Weeks	10 Weeks
SH	10.3 ± 0.4 (*n* = 31)	15.1 ± 0.6 ^##^ (*n* = 29)	20.3 ± 0.8 ^##^ (*n* = 25)	27.4 ± 1.1 ^##^ (*n* = 22)
LD	10.6 ± 0.4 (*n* = 32)	16.1 ± 0.6 ^##^ (*n* = 29)	21.8 ± 0.9 *^,##^ (*n* = 25)	29.6 ± 1.1 *^,##^ (*n* = 25)
RD	10.2 ± 0.3 (*n* = 32)	16.8 ± 0.5 *^,##^ (*n* = 31)	24.1 ± 0.8 **^,##^ (*n* = 29)	32.1 ± 0.9 **^,##^ (*n* = 29)
RT	10.4 ± 0.4 (*n* = 31)	16.5 ± 0.5 ^##^ (*n* = 30)	22.7 ± 0.8 *^,##^ (*n* = 26)	29.9 ± 1.0 **^,##^ (*n* = 25)

SH group, sham treatment; LD group, low-dose 0.05% GLZ addition; RD group, regular-dose 0.2% GLZ addition; and RT group, regular-dose antibiotic 0.2% Tilmicosin addition. Data are shown as mean ± SEM (** *p* < 0.01 and * *p* < 0.05 compared with the SH group, and ^##^ *p* < 0.01 compared with the age of 4 weeks group; two-way ANOVA followed by Student–Newman–Keuls multiple comparison posttest).

**Table 3:** Average daily body weight gain (kg) among four groups of weaned piglets.

Group/Age	4 Weeks	6 Weeks	8 Weeks	10 Weeks
SH	0.37 ± 0.06 (n = 31)	0.34 ± 0.07 (n = 29)	0.37 ± 0.09 (n = 25)	0.50 ± 0.09 ## (n = 22)
LD	0.37 ± 0.08 (n = 32)	0.39 ± 0.09 * (n = 29)	0.41 ± 0.08 *,## (n = 25)	0.56 ± 0.09 **,## (n = 25)
RD	0.36 ± 0.07 (n = 32)	0.47± 0.08 **,## (n = 31)	0.52 ± 0.08 **,## (n = 29)	0.57 ± 0.10 **,## (n = 29)
RT	0.37 ± 0.07 (n = 31)	0.43 ± 0.08 **,## (n = 30)	0.44 ± 0.07 **,## (n = 26)	0.51 ± 0.10 *,## (n = 25)

SH group, sham treatment; LD group, low-dose 0.05% GLZ addition; RD group, regular-dose 0.2% GLZ addition; and RT group, regular-dose antibiotic 0.2% Tilmicosin addition. Data are shown as the mean ± SEM (** *p* < 0.01 and * *p* < 0.05 compared with the SH group, and ^##^ *p* < 0.01 compared with the age of 4 weeks group; two-way ANOVA followed by Student–Newman–Keuls multiple comparison posttest).

**Table 4:** Average feed conversion ratio (%) among four groups of weaned piglets.

Group/Age	4 Weeks	6 Weeks	8 Weeks	10 Weeks
SH	30.1 ± 1.4 (*n* = 31)	31.3 ± 1.6 ^#^ (*n* = 29)	32.7 ± 2.9 ^#^ (*n* = 25)	33.4 ± 3.8 ^#^ (*n* = 22)
LD	30.2 ± 1.4 (*n* = 32)	32.8 ± 1.7 *^,#^ (*n* = 29)	33.8 ± 2.8 *^,#^ (*n* = 25)	34.6 ± 3.3 *^,#^ (*n* = 25)
RD	30.1 ± 1.3 (*n* = 32)	36.6 ± 1.6 **^,##^ (*n* = 31)	37.2 ± 2.8 **^,##^ (*n* = 29)	38.1 ± 3.0 **^,##^ (*n* = 29)
RT	30.1 ± 1.4 (*n* = 31)	33.1 ± 1.5 *^,#^ (*n* = 30)	34.1 ± 2.7 *^,#^ (*n* = 26)	35.9 ± 3.2 *^,#^ (*n* = 25)

SH group, sham treatment; LD group, low-dose 0.05% GLZ addition; RD group, regular-dose 0.2% GLZ addition; and RT group, regular-dose antibiotic 0.2% Tilmicosin addition. Data are shown as the mean ± SEM (** *p* < 0.01 and * *p* < 0.05 compared with the SH group, and ^##^ *p* < 0.01 and ^#^ *p* < 0.05 compared with the age of 4 weeks group; two-way ANOVA followed by Student–Newman–Keuls multiple comparison posttest).

**Table 5:** Average IgA, IgG, and IgE in the blood among four groups of weaned piglets.

Group/Age	SH	LD	RD	RT
IgA (ng/mL)	101.4 ± 23.5 (*n* = 3)	156.2 ± 27.1 ** (*n* = 3)	187.3 ± 26.2 ** (*n* = 3)	110.5 ± 24.1 (*n* = 3)
IgE (ng/mL)	233.7 ± 26.3 (*n* = 3)	171.6 ± 22.5 ** (*n* = 3)	134.5 ± 21.2 ** (*n* = 3)	221.4 ± 22.3 (*n* = 3)
IgG (μg/mL)	76.6 ± 17.1 (*n* = 3)	145.3 ± 25.3 ** (*n* = 3)	174.8 ± 27.6 ** (*n* = 3)	108.6 ± 21.2 * (*n* = 3)

SH group, sham treatment; LD group, low-dose 0.05% GLZ addition; RD group, regular-dose 0.2% GLZ addition; and RT group, regular-dose antibiotic 0.2% Tilmicosin addition. Data are shown as the mean ± SEM (** *p* < 0.01 and * *p* < 0.05 compared with the SH group; one-way ANOVA followed by Student–Newman–Keuls multiple comparison posttest).

## Data Availability

All the data are presented in this article.
